# Insights into the Mechanism Underpinning Composite Molecular Docking During the Self-Assembly of Fucoidan Biopolymers with Peptide Nanofibrils

**DOI:** 10.3390/md23040169

**Published:** 2025-04-15

**Authors:** Rui Li, Min-Rui Tai, Xian-Ni Su, Hong-Wu Ji, Jian-Ping Chen, Xiao-Fei Liu, Bing-Bing Song, Sai-Yi Zhong, David. R. Nisbet, Colin J. Barrow, Richard J. Williams

**Affiliations:** 1 Guangdong Provincial Key Laboratory of Aquatic Product Processing and Safety, Guangdong Province Engineering Laboratory for Marine Biological Products, Guangdong Provincial Engineering Technology Research Center of Marine Food, Key Laboratory of Advanced Processing of Aquatic Product of Guangdong Higher Education Institution, Guangdong Provincial Science and Technology Innovation Center for Subtropical Fruit and Vegetable Processing, Guangdong Provincial Engineering Technology Research Center of Prefabricated Seafood Processing and Quality Control, College of Food Science and Technology, Guangdong Ocean University, Zhanjiang 524008, China; tmr0168@163.com (M.-R.T.); suxn071@163.com (X.-N.S.); jihw62318@163.com (H.-W.J.); cjp516555989@gdou.edu.cn (J.-P.C.); liuxf169@126.com (X.-F.L.); song@gdou.edu.cn (B.-B.S.); 2 The Graeme Clark Institute, The University of Melbourne, Melbourne, VIC 3010, Australia; david.nisbet@unimelb.edu.au (D.R.N.); richard.williams@deakin.edu.au (R.J.W.); 3 Department of Biomedical Engineering, Faculty of Engineering and Information Technology, The University of Melbourne, Melbourne, VIC 3010, Australia; 4 Medical School, Faculty of Medicine, Dentistry and Health Science, The University of Melbourne, Melbourne, VIC 3010, Australia; 5 Centre for Sustainable Bioproducts, Deakin University, Waurn Ponds, VIC 3217, Australia; colin.barrow@deakin.edu.au; 6 College of Health Sciences, Abu Dhabi University, Abu Dhabi 59911, United Arab Emirates; 7 Institute for Mental and Physical Health and Clinical Translation, School of Medicine, Deakin University, Waurn Ponds, VIC 3217, Australia

**Keywords:** self-assembling peptides, electrostatic interaction, hydrogen bond, van der Waals, molecular docking simulation

## Abstract

Composite hydrogels with improved mechanical and chemical properties can be formed by non-covalently decorating the nanofibrillar structures formed by the self-assembly of peptides with fucoidan. Nevertheless, the precise interactions, and the electrochemical and thermodynamic stability of these composite materials have not been determined. Here, we present a thermodynamic analysis of the interacting forces that drive the formation of a composite fucoidan/9-fluorenylmethoxycarbonyl-phenylalanine-arginine-glycine-aspartic acid-phenylalanine (Fmoc-FRGDF) hydrogel. The results showed that the co-assembly of fucoidan and Fmoc-FRGDF was spontaneous and exothermic. The melting point increased from 87.0 °C to 107.7 °C for Fmoc-FRGDF with 8 mg/mL of added fucoidan. A complex network of hydrogen bonds formed between the molecules of Fmoc-FRGDF, and electrostatic, hydrogen bond, and van der Waals interactions were the main interactions driving the co-assembly of fucoidan and Fmoc-FRGDF. Furthermore, the sulfate group of fucoidan formed a strong salt bridge with the arginine of Fmoc-FRGDF. This study provides useful biomedical engineering design parameters for the inclusion of other highly soluble biopolymers into these types of hydrogel vectors.

## 1. Introduction

Molecular hydrogels are highly versatile materials with a diverse range of applications [1]. They are made from three-dimensional polymer networks that are cross-linked by covalent (chemical) or non-covalent (physical) interactions, absorbing and retaining large amounts of water and resisting dissolution. Self-assembling peptide (SAP) hydrogels are promising biomaterials as they are formed from the thermodynamically favored organization of individual molecules into nanofibrils that are readily programmed with ideal properties such as biocompatibility and multifunctionality [2]. Among short and minimalist peptide systems, Fmoc-functionalized peptide derivatives have emerged as useful low-molecular-weight gelators [3]. The unique properties of this system stem from the aromatic Fmoc moiety, which drives supramolecular interactions to enable spontaneous formation of nanofibrous hydrogels under physiologically benign conditions. The inherent self-assembly capability, coupled with tunable physicochemical properties, positions Fmoc-peptides as a versatile platform for biomaterial design.

Previously, we have shown that Fmoc-FRGDF undergoes spontaneous self-assemble into the nanofibrillar networks, yielding bioactive hydrogels in the absence of exogenous cross-linkers [4]. Nevertheless, hydrogels made from a single type of Fmoc-peptide exhibit significant limitations in biomedicine due to their inadequate mechanical properties and instability. To address these limitations, bioinspired strategies—mimicking natural extracellular matrices through multicomponent supramolecular systems—have emerged as a promising way to enhance functionality and stability. By co-assembling Fmoc-peptides with other (macro)molecules such as proteins [5] and polysaccharides [2], the mechanical properties of resultant hydrogels can be improved.

The complex sulfated polysaccharide fucoidan, also known as fucosan, is primarily extracted from marine brown algae and is characterized by its fucose content [6]. The significance of fucoidan in medicine, pharmaceuticals, and the food industry arises from its excellent water solubility, biodegradability, and biocompatibility, along with its notable bioactivities, which include immunomodulatory and other properties [7]. Additionally, as a natural marine polysaccharide, fucoidan can serve as a bioactive stabilizer for Fmoc-peptide hydrogels, enhancing their stability and improving mechanical properties [8]. It has been shown that the co-assembly of fucoidan with Fmoc-FRGDF peptide can form a supramolecular ordered structure, thus improving the mechanical properties of the composite hydrogel without affecting the π-β assembly [2]. More recently we have demonstrated fucoidan/Fmoc-FRGDF composite hydrogels as advanced delivery tools for hesperidin [9]. However, current research has predominantly focused on morphology and bioactivity associated with the two-component system. The effects of fucoidan on the electrochemical characteristics of composite hydrogels have not been thoroughly investigated. Additionally, there is limited understanding of the thermodynamic properties governing co-assembly, as well as the primary types of interactive forces and active sites between the two polymers.

In this study, we investigated the electrochemical properties (particle size and zeta potential) and thermodynamic properties (melting point and water distribution state) of fucoidan/Fmoc-FRGDF composite hydrogels. Molecular docking simulations and isothermal titration calorimetry (ITC) were employed to examine the interactions between fucoidan and Fmoc-FRGDF molecules. Our findings provide important design principles for the development of polysaccharide–protein stabilized hydrogels applicable to a wide range of fields, including tissue engineering, sensor technologies, cosmetics, personal care products, and the food industry.

## 2. Results and Discussion

### 2.1. Co-Assembly of Fucoidan and Fmoc-FRGDF to Form Composite Hydrogels

**Figure 1:**
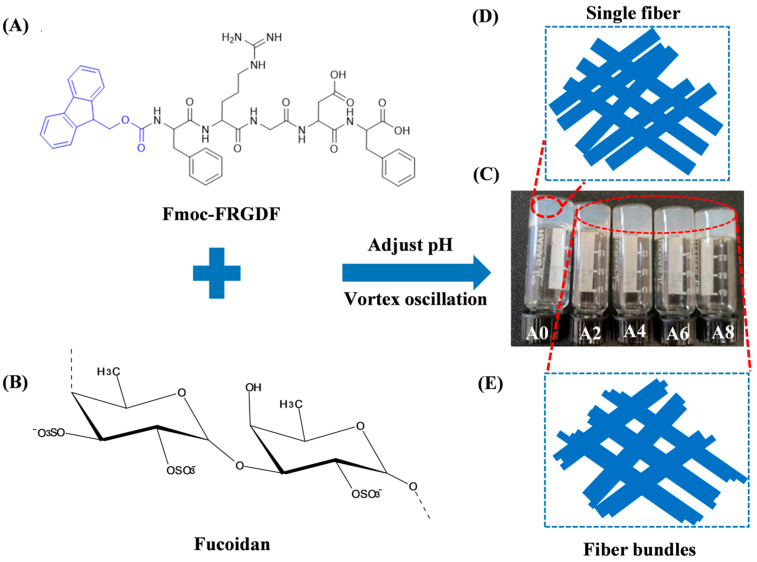
This schematic shows how fucoidan/Fmoc-FRGDF are co-assembled to form composite hydrogels. (**A**) Fmoc-FRGDF molecular formula (the blue moiety represents the Fmoc group); (**B**) fucoidan molecular formula; (**C**) A0–A8 hydrogel samples; (**D**) illustration of single nanofiber structure; and (**E**) illustration of multiple fiber bundle structure. A0 to A8 represent 10 mg/mL Fmoc-FRGDF hydrogels and composite hydrogels with fucoidan concentrations of 2, 4, 6 and 8 mg/mL, respectively.

The self-assembly system selected in this study was the amphiphilic aromatic peptide derivative Fmoc-FRGDF (Figure 1A). A simple self-assembling peptide hydrogel (A0), and composite hydrogels containing fucoidan (Figure 1B) at concentrations of 2, 4, 6 and 8 mg/mL (denoted as A2, A4, A6, and A8) were prepared using a pH switch method (Figure 1C). The pH of the hydrogel system was carefully adjusted to 7.4 to mimic the physiological conditions of the human body (pH ≈ 7.4). This intentional parameter selection was implemented to establish a biomimetic environment that simulates in vivo conditions, thereby enhancing the system’s biocompatibility for potential biomedical applications. At pH 7.4, the Fmoc group forms a skeleton through π-π stacking interactions, and the peptide side chains are connected via hydrogen bonds to form a stable β-sheet network, known as an π-β assembly. Following the stacking of these assemblies, nanotubes were formed (Figure 1D), which aligned to create bundles of nanofibers adorned with target peptide sequences. Fucoidan acted as a “glue”, binding individual nanofibers together and resulting in nanofiber bundles with increased stiffness (Figure 1E).

### 2.2. Particle Size, Zeta Potential, and Turbidity

Using electrochemical properties, particle size, zeta potential and turbidity measurements, we investigated the interactions between fucoidan and the self-assembling peptide Fmoc-FRGDF. The particle size distribution is closely associated with the texture of gels [10]. Generally, the particle size measured by dynamic light scattering reflects the hydrodynamic diameter, which is larger than the particle size of samples in their dry state [11]. Table 1 shows an average particle size was 748 ± 82 nm for the Fmoc-FRGDF hydrogel. A composite hydrogel with the smallest particle size of 609 ± 104 nm was obtained when the fucoidan concentration was 6 mg/mL. As the concentration of fucoidan increased to 8 mg/mL, the particle size of the composite hydrogel increased significantly, to 884 ± 171 nm, compared to that of A6 (*p* < 0.05). The analysis indicated that the average particle size of the co-assembly of fucoidan and Fmoc-FRGDF decreased with rising fucoidan concentration up to 6 mg/mL. This reduction in particle size is attributed to the formation of a denser structure as water molecules are expelled from the complex.

It has been found that polysaccharide-protein systems have a strong zeta potential that is influenced by the concentration of polysaccharides, pH, and ionic strength [12]. Table 1 shows that the pure Fmoc-FRGDF hydrogel had a strong negative charge of −31 mV at pH 7.4. Fucoidan was also negatively charged with a zeta potential of −26 ± 1 mV. The fucoidan/Fmoc-FRGDF composite system was significantly influenced by the strong negative charge of both polymers. With an increase in the fucoidan concentration to 6 mg/mL, the zeta potential decreased significantly from −31 ± 5 mV to −36 ± 2 mV (*p <* 0.05). However, with a continuous increase in fucoidan concentration to 8 mg/mL, the zeta potential of the composite hydrogel slightly increased to −34 ± 1 mV. This may be due to occurrence of saturation of the interactions between fucoidan and the amphiphilic SAP, resulting in some charges being masked by the hydrophobic groups of both polymers [13]. Electrostatic repulsion can effectively prevent intermolecular aggregation and precipitation when the absolute value of the zeta potential in a dispersion system exceeds 30 mV [14]. Consequently, the fucoidan/Fmoc-FRGDF composite hydrogel demonstrates thermodynamic stability. Moreover, fucoidan functions as a “glue”, binding individual nanofibers into more rigid bundles. By electrostatically interacting with SAP molecules, fucoidan contributes to the stability of the composite gel system. Adding additional polysaccharide did not significantly impact the system once it reached saturation with fucoidan.

A low polydispersity index (PDI) value indicates a more homogeneous system, with smaller values reflecting greater homogeneity. It has been reported that when the PDI value is lower than 0.3, the colloid disperses homogeneously [15]. Table 1 demonstrates that the PDI values of all samples exceeded 0.5, indicating the relative polydispersity of the hydrogel systems. Notably, the PDI values of the fucoidan/Fmoc-FRGDF composite hydrogel at fucoidan concentrations of 6 mg/mL were lower than those of the pure Fmoc-FRGDF system, indicating that the co-assembly of fucoidan with SAP enhanced the homogeneity of the composite hydrogels. Thicker bundles were formed (fucoidan-dominant) at higher fucoidan concentrations. Conversely, at lower concentrations, the microstructure remained unaffected (SAP-dominant), suggesting the existence of an optimal balance between the two components [16].

**Figure 2:**
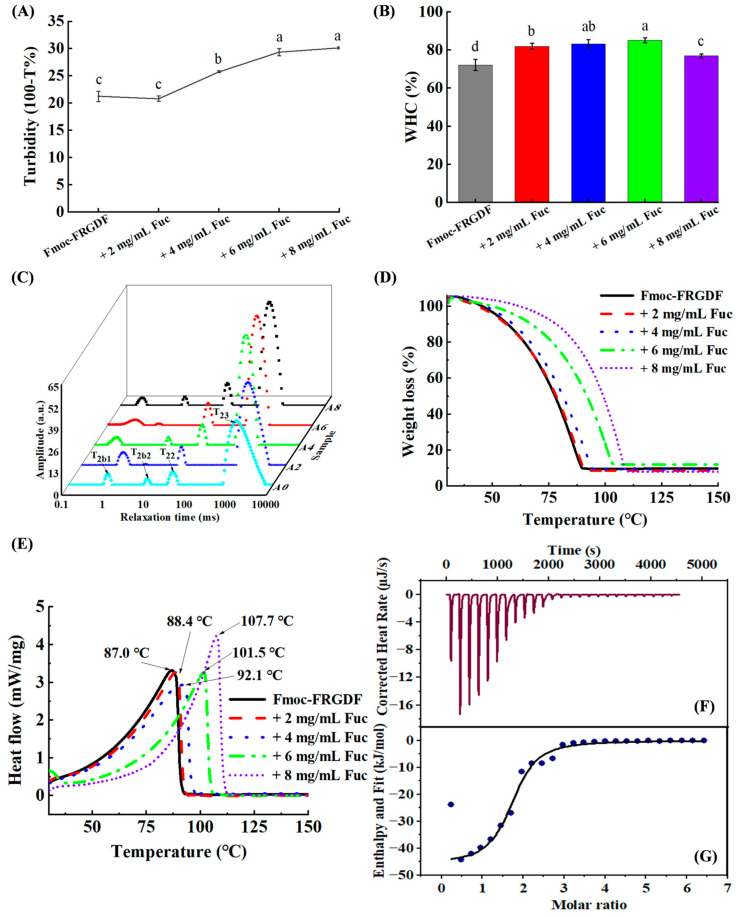
(**A**) Turbidity profile of composite hydrogels. (**B**) Water-holding capacity of composite hydrogel. (**C**) The self-relaxation time (T2) waterfall diagram measured by low-field nuclear magnetic resonance (LF-NMR) of composite hydrogel. (**D**) The thermogravimetric (TG), and (**E**) thermal stability (DSC) curves of composite hydrogel ranging from 30 °C to 150 °C at an increase rate of 2 °C/min under nitrogen environment. (**F**) Heat map and (**G**) binding isotherm of HCl solution titration of fucoidan (0.06 mg/mL) and Fmoc-FRGDF (0.1 mg/mL) mixed solution. Different letters in (**A**,**B**) indicate that the samples have significant differences (*p <* 0.05).

Next, turbidity measurement was used to determine the degree of protein aggregation. Figure 2A indicates that as the fucoidan content increases, the turbidity of the fucoidan/Fmoc-FRGDF composite hydrogel significantly increased (*p <* 0.05). This increase is attributed to the higher particle number per unit volume resulting from the greater fucoidan concentration. The presence of aggregates due to excessive fucoidan concentrations, or disordered co-assembly of the two polymer molecules, can also increase turbidity.

### 2.3. Water-Holding Capacity

The gel network structure was evaluated by its water-holding capacity (WHC). A low WHC value generally indicates an overall system with poor gel quality and low texture stability [17]. Figure 2B shows that the SAP-dominant Fmoc-FRGDF hydrogel had the lowest WHC. With the continuous increase in fucoidan, the WHC value gradually increased until the concentration of fucoidan increased to 4 and 6 mg/mL; the fucoidan-dominant WHC value was significantly higher than that of the other samples (83.28 ± 0.02% and 85.05 ± 0.01%, respectively; *p <* 0.05). These phenomena were due to the hydrophilic nature of fucoidan, where the hydroxyl group carried by fucoidan bound more water molecules through hydrogen bonds. This incorporated part of the water into the gel network, increasing the WHC of the gels. A dense gel network was formed by co-assembling fucoidan with self-assembled peptide nanofibers, as demonstrated by the microstructure of the gel networks [9]. The highly ordered and compact gel network structure provided more available space, enabling more water molecules to be locked in the gel network, thereby improving the WHC. Nevertheless, a further increase in fucoidan concentration to 8 mg/mL resulted in a decrease in water-holding capacity. The high concentration of fucoidan appeared to cause excessive aggregation, which ultimately led to the loss of water molecules.

### 2.4. Water State Analysis

Low-field nuclear magnetic resonance (LF-NMR) is a rapid, non-destructive and non-invasive method for water state analysis in food. It can obtain information about strongly bound water, bound water and weakly bound water by measuring relaxation time [18]. The water distribution of fucoidan/Fmoc-FRGDF composite hydrogels was analyzed by LF-NMR. The four peaks represented four different groups of water (T_2b1_, T_2b2_, T_22_ and T_23_) in the composite hydrogel (Figure 2C). T_2b1_ (0~1 ms) represents strongly bound water (very strongly bound to macromolecules), T_2b2_ (1~10 ms) indicates weakly bound water (strongly bound to macromolecules), T_22_ (10~100 ms) relates to immobile water (present in a dense network of fiber structures), and T_23_ (more than 100 ms) refers to free water (outside the network of fiber structures) [19].

Figure 2C and Table 2 indicate that free water accounts for the largest proportion in different water states and is the main form of water in composite hydrogels with different concentrations. In addition, compared with the relaxation time of pure SAP hydrogel, the T_23_ peak (free water) of fucoidan/Fmoc-FRGDF composite hydrogel was significantly shifted to the left (*p <* 0.05), which meant that the relaxation time T_23_ was shortened after the addition of fucoidan. Conversely, T_22_ had an increasing trend although there was no significant change (*p >* 0.05), indicating that the mobility of free water declined in the composite hydrogel network. Additionally, a shorter proton relaxation time promotes gel formation and counteracts dipole–dipole interactions [20]. As a result, the shortening of T_23_ can be attributed to better complexation between molecules after the addition of fucoidan. Due to the highly ordered structure, the hydrogel develops a denser microstructure, which explains why it becomes stiffer and more stable [21].

The percentage of peak area in different T_2_ intervals was compared to the total area and expressed as P_2_. With the increase in fucoidan concentration, the P_23_ area ratio of free water gradually decreased. When fucoidan was 6 mg/mL, the area ratio of P_23_ significantly decreased to 86.22% ± 0.28% from 88.63% ± 0.65% of sample A0 (*p <* 0.05) (Table 2). This indicated an increase in the non-flowing water area ratio (*p <* 0.05), which was attributed to the addition of fucoidan enhancing the binding of water molecules to multiple hydrophilic hydroxyl groups. As a result, a more compact and dense gel network was formed, limiting the movement of free water and resulting in immobilization of some free water in the gels matrix. The fucoidan interacted with water through hydrogen bonds, where some of the free water was immobilized [17]. Different water states behave differently in gels; thus, the stiffness and flexibility of hydrogels are more effectively improved by freezable bound water than by free water [22]. Consequently, in our system, the increased proportion of immobile water resulted in higher WHC and more stable composite hydrogels.

### 2.5. Thermal Stability

Thermogravimetric (TG) and thermal stability (DSC) of fresh hydrogels during the drying process were measured using a synchronous thermal analyzer. The TG curve in Figure 2D shows that the composite hydrogels have different weight loss with an increase in fucoidan concentration. The TG curves of the five samples were divided into two stages. In the first stage of 30–100 °C, the water in the surface and internal of all samples evaporated, and the weight decreased sharply with the increase in temperature. Due to different interactions between fucoidan, Fmoc-FRGDF, and water molecules, the slope of each curve decreased as the fucoidan concentration increased. During the 80~150 °C stage, the sample moisture was completely lost with the increase in temperature.

The DSC curve (Figure 2E) shows that all the sample curves had endothermic peaks. The maximum absorption peak (indicating the melting point) of the sample shifted to a higher temperature with the increase in fucoidan concentration, and the melting point increased from 87.0 °C of A0 to 107.7 °C of A8. This was attributed to the addition of fucoidan inducing more hydrogen bonds and improved thermal stability of the composite hydrogels [23]. This result indicated that the co-assembly of fucoidan with SAP can improve the stability of the resultant hydrogel. A similar study conducted by Wee et al. demonstrated that the addition of negatively charged soy polysaccharides to lactoferrin improved its thermal stability. The enhanced thermal stability of the composite nanogels is likely attributed to the formation of hydrogen bonds and electrostatic interactions [24]. Furthermore, the incorporation of fucoidan enhances the binding capacity of water molecules within the polymer network due to the large number hydroxyl and carboxyl groups in the fucoidan structure. This increase in bound water content results in a significant elevation in endothermic peak temperature [25].

### 2.6. Thermodynamic Properties

Isothermal titration calorimetry (ITC) is an effective method for calculating thermal changes and characterizing how molecules interact. ITC can provide thermodynamic parameters, such as binding ratio (n), enthalpy changes (ΔH), entropy changes (ΔS), binding constants (K) and Gibbs free energies (ΔG) [26]. Gibbs free energy can be calculated by the equation ΔG = ΔH − TΔS. These parameters have been widely used to characterize the interaction between biological macromolecules. The temperature diagram (heat rate—time curve) obtained by the titration of fucoidan/Fmoc-FRGDF mixed solution with HCl at pH 7.4 and 25 °C is shown in Figure 2F,G, and the multiple-site model used to calculate the reaction parameters is shown in Table 3.

Generally, if ΔG is less than 0, the reaction is considered spontaneous. Table 3 shows that during the binding reaction of fucoidan and Fmoc-FRGDF, TΔS > ΔH, which determined that ΔG (=ΔH − TΔS) < 0. According to these results, the fucoidan/Fmoc-FRGDF interaction at room temperature (298 K) was spontaneous, indicating that the electrostatic binding between two natural biopolymers is spontaneously exothermic [27]. The four major non-covalent interactions between molecules are electrostatic interaction, hydrogen bonding, hydrophobic force and van der Waals force. The highly negative value of ΔH indicated that the predominant type of interaction is electrostatic [26]. Additionally, the negative values of ΔH, and ΔS reflected the contributions of van der Waals interactions and hydrogen bond formation in low-dielectric media (Table 3) [28].

### 2.7. Molecular Dynamic Simulation of Peptide Self-Assembly

Molecular docking is a powerful tool for visualizing the interactions between receptors and ligand molecules, such as proteins, polysaccharides and polyphenols [29]. By calculating the degree of structural matching between the two polymers, molecular simulation and molecular docking of reactants were carried out, through which we can understand the reaction sites and the corresponding force types [30], and determine how small molecules interact with biological macromolecules, including binding sites, binding modes, and amino acid residues involved.

**Figure 3:**
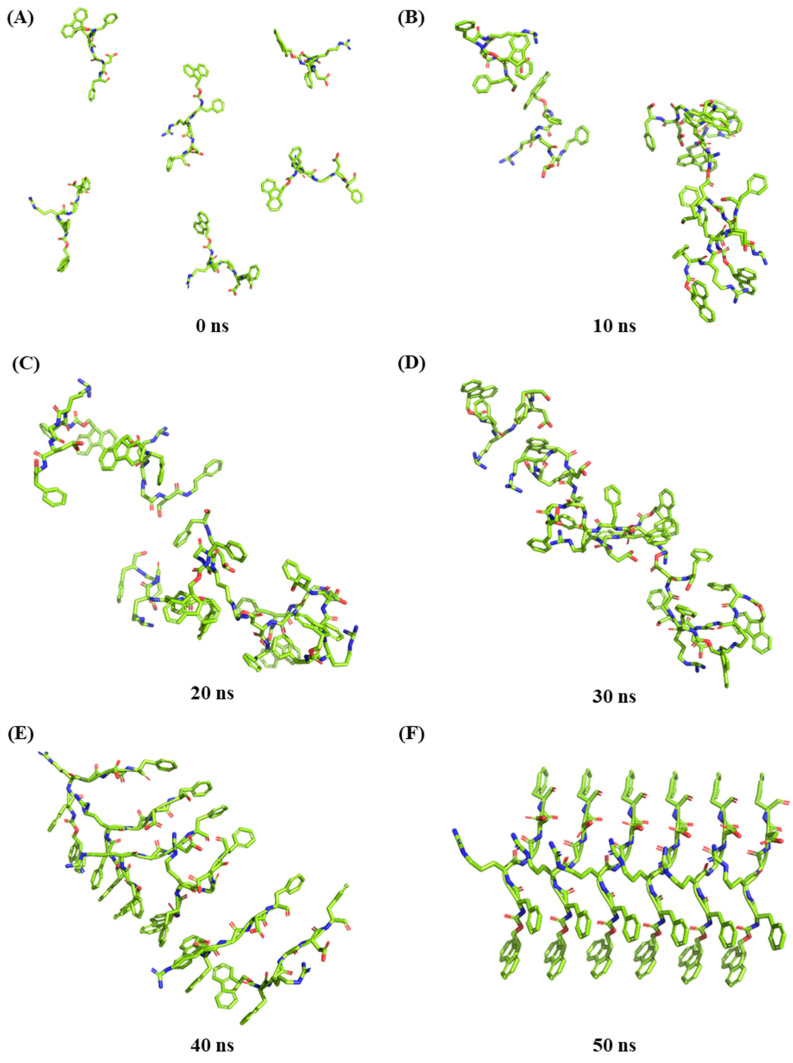
The three-dimensional structure of the dynamic simulation of six Fmoc-FRGDF molecules self-assembly at different times. (**A**) 0 ns, (**B**) 10 ns, (**C**) 20 ns, (**D**) 30 ns, (**E**) 40 ns and (**F**) 50 ns.

**Figure 4:**
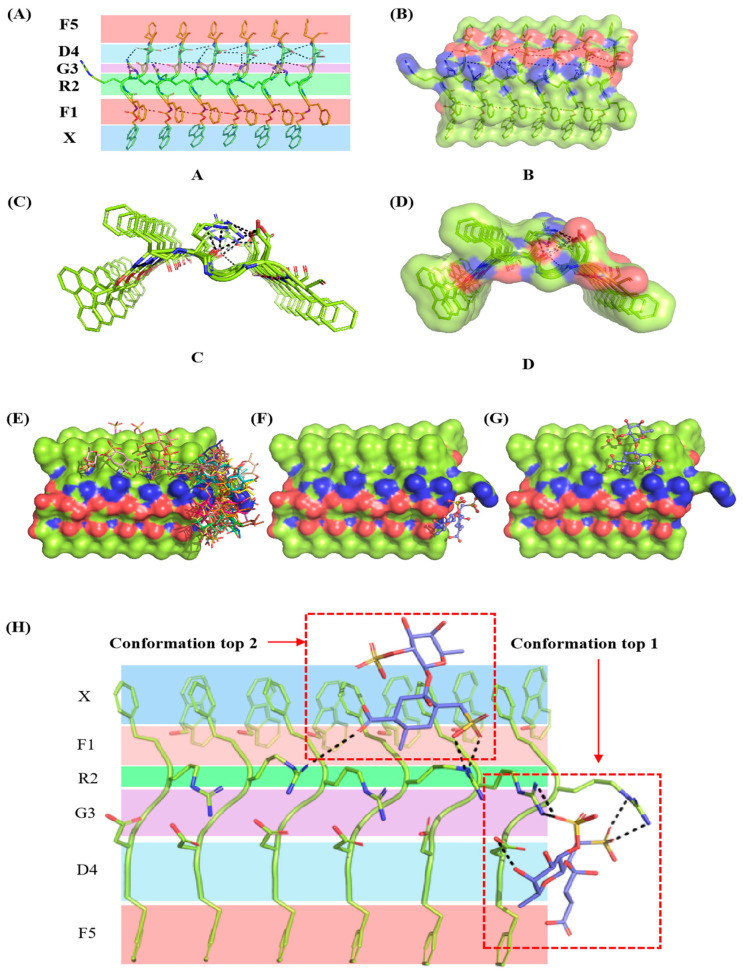
The three-dimensional crystalloid structure of Fmoc-FRGDF self-assembly simulation and the three-dimensional molecular docking conformations of fucoidan and Fmoc-FRGDF according to the score of their binding energy. The picture (**A**), and the surface picture (**B**), the longitudinal view (**C**), and the surface longitudinal view (**D**) of the amino acids and their grafting sites of Fmoc-FRGDF. X: Fmoc, F1: Phenylalanine, R2: Arginine, G3: Glycine, D3: Aspartic acid, F5: Phenylalanine (the dotted line represents hydrogen bond). (**E**) The predicted top 50 conformations. (**F**) Top 1. (**G**) Top 2. The black dotted line showed the formed salt bridge. (**H**) The molecular structures of the binding sites of Fmoc-FRGDF with fucoidan for conformations 1 and 2.

In this study, peptide self-assembly simulation was carried out due to the complexity of the combine peptide self-assembled and concurrent interact with fucoidan. Six peptide molecules were randomly positioned in lattice boxes for dynamic simulation and analysis, ultimately resulting in the formation of a crystalloid structure (Figure 3). The simulation analysis showed that the peptide molecules stacked directly on each other. The benzene rings of different molecules formed a stable aromatic stack structure between the plane rings of Fmoc-FRGDF molecules via π-π effects, confirming our previous mechanistic hypothesis [4,9,16]. The hydrogen bond is one of the most important molecular interactions in biology that could provide selectivity to protein–ligand interactions [31]. Figure 4A–D shows that the positively charged arginine amino acids and the negatively charged aspartic acid amino acids of the Fmoc-FRGDF molecules form relatively complex polar hydrogen bond networks. Molecular dynamic simulations showed strong interactions between the peptide molecules [32]. A stable aromatic stacked structure between the planar rings of peptides was also formed between the grafted functional groups of the head (the fifth phenylalanine) (Figure 4A–D).

### 2.8. Binding Mode Analysis of Fucoidan

A molecular docking study of fucoidan’s binding mode to Fmoc-FRGDF was conducted after molecular dynamic simulations of the peptide molecules were completed. In total, the top 50 systems were chosen according to their energy score to predict their inaction sites (Figure 4E), and the top 2 systems were selected (Figure 4F,G). Although the binding sites of conformation top 1 and top 2 were inconsistent, their binding energy was almost equal, −32.57 kJ/mol of conformation top 1 and −31.48 kJ/mol of top 2. The sulfuric acid radical group of fucoidan had a strong electronegativity, which could form a strong salt bridge (black dotted linen in Figure 4H) with the extremely positive arginine amino acids and play a key role in the binding between the two molecules (Figure 4H).

An autoDock 4.2 hybrid approach was used to calculate the binding energy between fucoidan and Fmoc-FRGDF, as well as other non-bonded interactions (Table 4). The non-bonded interactions, such as van der Waals energy (ΔG_vdw_), H-bond energy (ΔG_H-bond_), and electrostatic energy (ΔG_ele_), governed the total binding energy (ΔG_bind_). Across all types of intermolecular forces, the electrostatic energy ΔG_ele_ was −25.70 kJ/mol of conformation top 1 and −23.86 kJ/mol of conformation top 2 contributed the most to the average binding energy in the fucoidan–peptide interaction, followed by the hydrogen bond energy ΔGH-bond, −10.13 kJ/mol and −8.62 of conformation top 1 and top 2, respectively. The extent of desolvation for any ligand atom varies between different ligand conformations due to its dependence on the position of other ligand atoms [33]. Consequently, the desolvation energies were calculated to be −4.90 kJ/mol for conformation top 1 and −7.70 kJ/mol for conformation top 2. The intermolecular energy (ΔG_int_) is the sum of ΔG_vdw_, ΔGH-bond, ΔG_desol_ and ΔG_ele_. The values of ΔG_int_ were −48.81 kJ/mol for conformation 1 and −47.72 kJ/mol for conformation 2, respectively. The torsional energy (ΔG_tor_), which evaluates the strain of a molecule’s conformation, exhibited unfavorable energy contributions that opposed binding. Typically, a lower total binding energy (ΔG_bind_) correlates with a higher binding efficiency and a strong interaction [32]. Specifically, when the ΔG_bind_ falls below −29.3 kJ/mol, the polymer demonstrates a strong binding force [34]. The ΔG_bind_ values for conformation tops 1 and 2, after incorporating torsional energy, were −32.57 kJ/mol and −31.48 kJ/mol, respectively, indicating high binding stability for both docking complexes. Conversely, a lower K_d_ value signifies greater stability of the system. The binding affinity K_d_ between the two molecules were found to be 1.93 µmol/L for conformation top 1 and 2.99 µmol/L for conformation top 2 (ΔG = RTlnK_d_), further demonstrating the formation of a stable system. These results suggest that the hybrid methods of AutoDock 4.2 validate the binding energy estimates obtained from molecular docking, and molecular dynamics reveal that the co-assembly of fucoidan and Fmoc-FRGDF is spontaneous, primarily mediated by electrostatics, van der Waals force, and hydrogen bond interactions. These findings are consistent with the results obtained from ITC.

## 3. Materials and Methods

### 3.1. Material

*Undaria pinnatifida* fucoidan was purchased from Qingdao Mingyue Seaweed Group (Qingdao, China) with a molecular weight of 239.09 kDa, total sugar content of 89.09% (*w*/*w*), sulfate content of 42.30% (*w*/*w*), and glucuronic acid content of 16.70% (*w*/*w*). The monosaccharides comprise L-fucose, D-xylose, D-glucose, D-glucuronic acid, and D-Mannose with a molar percentage of 56.61:36.15:3.61:2.00:1.63. Fmoc-FRGDF (purity > 95%, and molecular weight 863 Da) was purchased from Pepmic Co., Ltd. (Suzhou, China). In addition, 0.1 M HCl, 0.5 M NaOH, and 0.1 M potassium phosphate buffer (PBS, pH 7.4) and other chemicals were all analytical grade.

### 3.2. Preparation of Fucoidan/Fmoc-FRGDF Composite Hydrogels

The hydrogel preparation method was in accordance with our previously used method [9]. Specifically, 0, 2, 4, 6, and 8 mg fucoidan and 10 mg Fmoc-FRGDF were weighed into a 4 mL glass vial, respectively. Then, 400 μL distilled water, 65 μL 0.5 M NaOH solution were added in turn until completely dissolved, followed by the dropwise addition of 0.1 M HCl solution to neutralize the system to pH 7.4. Finally, 0.1 M PBS solution (pH = 7.4) was added into the solution to make the total volume 1.0 mL. All procedures were carried out on a vortex oscillator. Finally, air bubbles were removed using sonication. The cocktail was kept in an ambient environment to form hydrogels.

### 3.3. Particle Size and Zeta Potential

The gel samples were diluted with deionized water to 1.0 mg/mL (based on peptide weight) and mixed evenly. The zeta potential and particle size were measured using a Malvern Zetasizer Nano ZS90 (Shanghai Malvern Panaco Co., Ltd., Shanghai, China), with phase analysis light scattering (PALS) and dynamic light scattering (DLS), respectively. All gels were scanned three times at 25 °C.

### 3.4. Turbidity

The gel samples were diluted to 4 mg/mL (based on peptide weight) using deionized water. The absorbance was recorded at 600 nm using a UV-1700 spectrophotometer (Shimadzu, Kyoto, Japan). The absorbance was converted into turbidity using the formula (100%-T%) (T is the transmittance), and deionized water was used as the blank control. All samples were scanned three times at 25 °C.

### 3.5. Determination of Water-Holding Capacity

The gel samples (1.0 mL) were weighed into a centrifuge tube and recorded as M1. They were then centrifuged at 12,000 r/min for 20 min at 25 °C using a high-speed frozen centrifuge (Universal 320R, Hettich, Tuttlingen, Germany). After removing the upper liquid, the samples were weighed again and recorded as M2. Finally, the water-holding capacity (WHC %) of the gel sample was calculated using Equation (1) [35].

WHC (%) = M2/M1 × 100%(1)

### 3.6. Synchronous Thermal Analyzer Analysis

The fresh gel samples were placed into an aluminum crucible, and the thermal stability of fucoidan/Fmoc-FRGDF hydrogels with different fucoidan concentrations was determined by a synchronous thermal analyzer (STA449F3, Netzsch, Selb, Germany). The heating rate was 2 °C/min, and the temperature was 30~150 °C.

### 3.7. Low-Frequency Nuclear Magnetic Resonance Analysis

The water distribution state of the gels was determined using low-frequency nuclear magnetic resonance spectroscopy, using an NMI 20-060H-I NMR analyzer (Niumag Co., Ltd., Suzhou, China). The stable frequency operation was performed at 32 °C with a 21 MHz, 60 mm coil. Fucoidan/Fmoc-FRGDF composite hydrogel samples were subjected to T2 relaxation scanning. The scans were repeated 3 times, and the number of echoes was 8000 times. The transverse relaxation time T2 of the samples was collected by the Carr–Purcell–Meiboom–Gill (CPMG) pulse sequence, and the relaxation time of the T2 sample was obtained using the synchronous iterative reconstruction technique to invert the data [36].

### 3.8. Isothermal Titration Calorimetry

The interactions between fucoidan and Fmoc-FRGDF were analyzed using a Nano ITC isothermal titration calorimeter (TA Instruments, New Castle, DE, USA). The concentrations of fucoidan solution and Fmoc-FRGDF solution were 0.06 mg/mL and 0.1 mg/mL, respectively. Both were then mixed at a ratio of 1:1 (*v*/*v*). Then, 4.75 mM HCl solution was used to titrate the sample pool to make the end point pH at 7.4. At the same time, NaOH solution was titrated with HCl as the control group. The mixture was kept at 25 °C without stirring. During data processing, the reaction heat generated by the titration of NaOH solution with HCl was measured and subtracted from the original data to determine the corrected enthalpy change. The thermodynamic parameters, dissociation constant Kd (reciprocal of binding constant Ka), stoichiometric ratio n, enthalpy change ΔH and entropy change ΔS were obtained by fitting the data using the multiple-site model. The Gibbs free energy change (ΔG) was calculated by the equation (ΔG = ΔH − TΔS).

### 3.9. Molecular Simulation

A large-scale atomic/molecular massively parallel simulator (LAMMPS) software (29Sep2021-para, Sandia National Laboratories, Albuquerque, America) was used to simulate the molecular dynamics of peptide self-assembly. The molecular dynamic simulation process was as follows: (1) Two-step energy minimization. The energy of the water molecules was minimized in the first cycle, and the energy of the entire system was minimized in the second cycle for 5000 times each. (2) System balance. The first was the temperature balance process of the system. The Langevin temperature control method was used to balance 100 ps to raise the temperature of the system from 0 °C to 38 °C. Then, the isotropic Berendsen pressure control method was used to carry out the pressure boost balancing process for 100 ps. (3) Dynamic simulation. It is an unlimited free simulation stage. In the kinetic process, a constant temperature of 38 °C was used to simulate the molecular dynamics of the whole system. The cut-off distance of van der Waals energy and short-range electrostatic energy was 10 Å, and the long-range electrostatic energy was calculated by the Particle-mesh Ewald (PME) method. (4) In the process of molecular dynamics, the molecular dynamics time was controlled at 50 ns, and the average structure was used for subsequent binding mode analysis.

### 3.10. Molecular Docking

The Fmoc-FRGDF self-assembled crystalline protein was designated as the receptor, while the fucoidan molecule served as the ligand. The AutoDock Tools 1.5.6 was utilized to perform a conformational search and evaluate the ligand based on the scores derived from systems with varying conformations, orientations, positions, and energies. A total of 200 molecular conformations were docked, and the clustering analysis module was employed to rank them according to their molecular docking energy scores. From this analysis, the top 50 systems with the highest clustering and lowest energy scores were selected for further examination of their interaction sites. Ultimately, the top two complex systems exhibiting the maximum clustering and minimum energy were chosen for a detailed analysis.

### 3.11. Statistical Analysis

Three parallel experiments were performed with at least triplicate samples within each analysis. The results were shown in the form of the mean ± standard deviation (SD). One-way analysis of variance (ANOVA, *p* < 0.05) and Duncan’s multiple range test were carried out to analyze the differences between samples using Statistic Package for the Social Science software (SPSS 25.0, SPSS Inc., Chicago, IL, USA).

## 4. Conclusions

The self-assembling peptide Fmoc-FRGDF is effective at hydrogelation, but it has poor mechanical properties, limiting its applications in biomedicine to ‘soft’ applications. We have previously shown that the co-assembly of fucoidan and Fmoc-FRGDF enhances the mechanical properties of a Fmoc-FRGDF peptide hydrogel. However, the exact interaction forces were not clear. Based on isothermal titration calorimetry and molecular docking techniques, this study attempted to answer this question from a thermodynamic perspective. The results showed that when the fucoidan concentration was at 6 mg/mL, the smallest average particle size (609 ± 104 nm), maximum turbidity and water-holding capacity could be obtained. Furthermore, we investigated the change in water state of the fucoidan/Fmoc-FRGDF composite hydrogel for the first time. The co-assembly of fucoidan with Fmoc-FRGDF caused the free water (P_23_) to move towards the immobile water (P_22_), resulting in a denser and more stable structure of the composite hydrogel, which explained the enhanced water-holding capacity and heat resistance of the fucoidan/Fmoc-FRGDF composite hydrogel. The melting point increased from 87.0 °C of A0 to 107.7 °C of A8. The benzene rings of different molecules formed a stable aromatic stack structure between the plane rings of Fmoc-FRGDF molecules via π-π assembly. The main interactions were hydrogen bonding, electrostatic interactions, and van der Waals forces. Fucoidan and SAP action sites have, for the first time, been simulated using molecular docking. The insights presented in this work can inform the design of new biomaterials using the extensive library of polysaccharides and peptides available in nature.

## Figures and Tables

**Table 1:** Particle size, zeta potential and polydispersity index (PDI) of A0, A2, A4, A6, A8, and fucoidan solution.

Sample	Particle Size (nm)	Zeta Potential (mV)	PDI
A0	748 ± 82 ^ab^	−31 ± 5 ^ab^	0.81 ± 0.20 ^ab^
A2	896 ± 89 ^a^	−39 ± 1 ^c^	0.96 ± 0.04 ^a^
A4	851 ± 170 ^a^	−37 ± 3 ^c^	0.80 ± 0.17 ^ab^
A6	609 ± 104 ^b^	−36 ± 2 ^c^	0.65 ± 0.11 ^bc^
A8	884 ± 171 ^a^	−34 ± 1 ^bc^	0.77 ± 0.08 ^abc^
Fucoidan solution	522 ± 24 ^b^	−26 ± 1 ^a^	0.54 ± 0.07 ^c^

Note: A0–A8: fucoidan (0, 2, 4, 6, 8 mg/mL)/Fmoc-FRGDF (10 mg/mL) hydrogel; fucoidan solution: 5 mg/mL fucoidan. Different letters in each column indicated significant difference at *p <* 0.05.

**Table 2:** LF-NMR self-relaxation times (T_2_) and peak area (P_2_) of fucoidan/Fmoc-FRGDF hydrogels.

Sample	Distribution of Relaxation Time T_2_/ms				Proportion of Relaxation Time Peak Area P_2_/%			
	T_2b1_	T_2b2_	T_22_	T_23_	P_2b1_	P_2b2_	P_22_	P_23_
A0	0.91 ± 0.02 ^a^	8.4 1± 0.06 ^a^	36.22 ± 0.17 ^b^	1320 ± 13 ^a^	3.69 ± 0.59 ^c^	1.84 ± 0.57 ^ab^	5.76 ± 0.21 ^b^	88.63 ± 0.65 ^a^
A2	1.01 ± 0.03 ^a^	3.71 ± 0.58 ^c^	30.85 ± 1.58 ^b^	1154 ± 7 ^b^	6.09 ± 0.27 ^a^	0.36 ± 0.13 ^c^	5.66 ± 0.34 ^b^	88.11 ± 0.08 ^a^
A4	0.37 ± 0.01 ^b^	7 ± 1 ^b^	45 ± 6 ^a^	505 ± 4 ^c^	4.66 ± 0.68 ^b^	1.62 ± 0.40 ^b^	6.94 ± 0.34 ^a^	86.59 ± 1.08 ^b^
A6	0.48 ± 0.09 ^b^	1.90 ± 0.62 ^d^	30 ± 1 ^b^	471 ± 9 ^d^	4.65 ± 0.39 ^b^	0.65 ± 0.26 ^c^	7.47 ± 0.48 ^a^	86.22 ± 0.28 ^b^
A8	0.38 ± 0.20 ^b^	3.81 ± 0.14 ^c^	47 ± 5 ^a^	433 ± 6 ^e^	3.65 ± 0.42 ^c^	2.51 ± 0.45 ^a^	7.41 ± 0.39 ^a^	86.95 ± 0.18 ^b^

Note: The content of fucoidan is 0, 2, 4, 6, and 8 mg/mL, respectively, and the content of Fmoc-FRGDF is 10 mg/mL. Different letters in the same column indicate significant differences *(p <* 0.05).

**Table 3:** The Isothermal Titration Calorimetry binding parameters of fucoidan and Fmoc-FRGDF in HCl solution at 25 °C.

Binding Ratio (n)	K_d_ (µM)	ΔH (kJ/mol)	ΔS (kJ/mol)	TΔS (kJ/mol)	ΔG (kJ/mol)
1.68	6.66	−45.80	−0.050	−16.22	−29.57

**Table 4:** Combined free energy of fucoidan and Fmoc-FRGDF molecules.

Items	Conformation Top 1	Conformation Top 2
ΔG_vdw_	−8.08 kJ/mol	−7.53 kJ/mol
ΔG_H-bond_	−10.13 kJ/mol	−8.62 kJ/mol
ΔG_desol_	−4.90 kJ/mol	−7.70 kJ/mol
ΔG_ele_	−25.70 kJ/mol	−23.86 kJ/mol
ΔG_int_	−48.81 kJ/mol	−47.72 kJ/mol
ΔG_tor_	16.24 kJ/mol	16.24 kJ/mol
ΔG_bind_	−32.57 kJ/mol	−31.48 kJ/mol

Note: ΔG_vdw_, ΔG_H-bond_, ΔG_desol_, ΔG_ele_, ΔG_Int_, ΔG_tro_, and ΔG_bind_ refer to van der Waals force, hydrogen bond energy, desolvation energy, electrostatic energy, intermolecular energy, torsional energy, and total binding energy, respectively. ΔG_Int_ = ΔG_vdw_ + ΔG_H-bond_ + ΔG_desol_ + ΔG_ele_; ΔG_bind_ = ΔG_in_t + ΔG_tor_.

## Data Availability

Data are available upon request from the corresponding authors.
